# Kornai on the affinity of systems: Is China today an illiberal capitalist system or a communist dictatorship?

**DOI:** 10.1007/s11127-020-00835-0

**Published:** 2020-07-27

**Authors:** Péter Mihályi, Iván Szelényi

**Affiliations:** 1 Department of Economics, Corvinus University of Budapest and Central European University, Budapest, Hungary; 2 Yale University, New Haven, USA

**Keywords:** China, Liberalism, Illiberalism, Dictatorship, Comparative economics, B14, P11, P14, P21

## Abstract

More than 40 years ago, János Kornai introduced his famous supermarket metaphor. Socioeconomic systems cannot be constructed from purposely selected features, similar to customers in a supermarket, who can freely put into their shopping trolley whatever they like. Systems constitute an organic whole. They contain good and bad features in fixed proportions. After 1990, Kornai and most Western commentators expected that as market integration and private property expand, China would eventually turn into a liberal democracy. Prior to the worldwide fall of communism, Kornai had three primary criteria to determine whether a country was socialist or capitalist; later he amended this with six secondary ones. The present paper introduces into this list an additional 11 criteria—i.e. 20 quantifiable metrics altogether. Kornai was among the very first to recognize that with President Xi Jinping taking charge, China made a U-turn. While capitalist elements remain strong, in the final analysis, the country is on its way back to where it was before 1978.

## Introduction

In 1979, János Kornai introduced his famous supermarket metaphor to challenge the theory of the “third way”, an *optimal* and *sustainable hybrid* between capitalist and communist policies. Socioeconomic systems, he argued, cannot be constructed from randomly or scientifically selected pieces, similar to customers in a supermarket, who are free to put into their shopping trolley whatever they find on the shelves. Structures like socialism^1^ or capitalism are not made of separate building blocks fixed together with screws or mortar. Their interrelatedness is like a genetic program of procreation. All newborn cats are similar to each other, whether small or big. In the same way, all socialist (or capitalist) systems are somewhat similar to each other. In several later works, Kornai formulated the same idea as the “affinity thesis”,^2^ according to which the bureaucratic model of coordination has a natural affinity for (*strong* linkage to) state-owned property, while market coordination has a natural affinity for private property. By contrast, the linkage between market coordination and state property is *weak*, meaning that one cannot use the market as a neutral instrument to promote state ownership.^3^

The objective of the present paper, based partly on our new book (Szelenyi and Mihályi 2020b), is to further advance Kornai’s affinity thesis on the example of China, about which Kornai himself has published quite extensively and spoken often in interviews since 2014. Kornai’s initial position was that China was a *capitalist dictatorship*. The Communist Party was communist only in its name, he asserted; it was more nationalist or Confucian.^4^ In the present paper, by contrast, we shall elaborate on Kornai’s more recent position (Kornai 2014, 2019a). China between 1978 and 2013 resembled non-electoral, authoritarian capitalist regimes like Russia, Iran, or the Gulf monarchies. Elections are held, but the political rulers manipulate the electoral system in many ways, so the outcome is not competitive. It is always the incumbent ruler who wins. Present-day China, under the leadership of Xi Jinping, is increasingly returning to *communist*, *dictatorial practices*. The regime retains and even extends governmental interference into markets and private property (which was substantial even in the earlier phases of the reform).^5^ In the light of Kornai’s supermarket metaphor, such a system may become unsustainable. Whether it is untenable or not remains to be seen. Only history will tell, but Kornai’s theory predicts eventual failure.

At the time of submitting the final manuscript of the present essay (May 2020), when the entire world is occupied with the fight against the coronavirus, such a “categorization game” is very relevant for the interpretation of President Xi’s “people’s war against the epidemic with the most comprehensive and rigorous measures”.^6^

## The end of history?

In a path-breaking article, the American political scientist Francis Fukuyama (1989) predicted “the end of history” and the *final victory of the Western-type liberal, capitalist system*. He wrote it in a few months between November 1989 (the collapse of the Berlin Wall) and the beginning of Boris Yeltsin’s rule in July 1991 (as president of the newly “re-created” Russia). But history continued. Fukuyama’s point of departure was the fall of communism, the only historically proven, 20th century viable alternative to the capitalist market economy and liberal political systems. (Fascism, the other abysmal alternative to liberal democracies, proved to be relatively short-lived from the 1920s until the 1970s.)^7^

Indeed, as Szelenyi and Mihályi (2020b) explained, 70 years after Russia’s Bolshevik Revolution, 26 socialist countries spanned more than 31% of four continents. Their combined populations in 1987 amounted to 34% of the world’s total. Then, unexpectedly on 9 November 1989, half a million people gathered in East Berlin in a mass protest; the Berlin Wall dividing communist East Germany from capitalist West Germany crumbled. Within a few months, the communist system disappeared from Europe, and the two Germanys were united. And, indeed, the first 15 years following the publication of Fukuyama’s seminal article saw a spectacular retreat of socialist systems worldwide and the expansion of freedom and liberal democracies. The most persuasive argument for an optimistic interpretation of ongoing history was the relatively peaceful disintegration of the Soviet Union (and with that the liberation of the three Baltic states), but the changes in China offered the second definitive proof. Although five other countries (Cambodia, Cuba, Laos, North Korea, and Vietnam) have remained faithful to communist principles, those countries were usually relegated to footnotes as actually existing but quantitatively insignificant counterexamples. It is appropriate to treat the four Asian countries as China’s satellites—which in fact they are. (Cuba is a special case.)^8^

Size matters: The Union of Soviet Socialist Republics (USSR) was and the People’s Republic of China (PRC) is a global superpower. Had the Soviet-type planned economy not been abandoned by the USSR and its allies in Eastern Europe, and the socialist world system remained a more or less credible alternative to the capitalist market economy, China, or even India and Brazil—i.e., the largest and politically strongest states of the “third world”—might not ever have opened their markets to Western multinational companies to the extent that they did. Thus, globalization would not have happened either—at least not with the same speed as it did happen.

It is well known that the Chinese economic reforms arising from the dismal performance of the classical socialist model started earlier, in 1978. However, what matters from the perspective of this paper is that the reforms continued after 1989. In 1978, the term “reform” referred merely to modernization, more specifically the “Four Modernizations Program” of Deng Xiaoping (Shi 1998, p. 5). In name at least, reform referred mainly to the reopening of the 1963 modernization program associated with then Prime Minister Zhou Enlai. Very importantly, in the new interpretation, that program included—in fourth place—the Open Door Policy, an effort to attract foreign capital. So the language changed slowly, although Yu Guangyuan supposed that Deng might have used the term “market economy” as early as 1979.^9^ The expression “market economy” only began to appear in academic publications by the mid-1980s (more cautious Chinese authors preferred to write about a “commodity economy”). In 1984, the official party line was, “We do not practice the market economy which is completely regulated by the market”, phraseology ambiguous enough to be used by both those who supported and those who opposed the market economy, in order to argue that their position was backed by the party (Yu 2005, p. 37).

Deng Xiaoping was one of the most complex and consequential politicians of the 20th century. He joined the Communist Party of China (CPC) in 1924 when he was just 20 years old, fought with the communists against the Kuomintang and Japanese, and was rewarded with a vice-premiership in 1952. His main allies were the reformer Zhou Enlai and Liu Shaoqi. Deng opposed including “Mao’s thoughts” in the Chinese constitution;^10^ after the failure of the Great Leap Forward, he, together with Zhou and Li, tried to rebuild the economic institutions destroyed by Mao. Unsurprisingly, at the start of the Cultural Revolution in 1966, he lost all of his positions. After Mao’s death in 1976, Deng came back. He outsmarted his conservative enemies (the so-called Gang of Four) and by 1978 was China’s de facto ruler.^11^

The post-1978 reforms were not merely a reincarnation of the Four Modernizations Program. Given China’s cultural respect for tradition, Deng realized that he could not break with Mao like Khrushchev and Gorbachev had rejected Stalin. Hence, Deng’s famous verdict on Mao’s historical role: “He was 70 per cent good and 30 per cent bad”. Whatever we know about him today, Deng was hardly a communist ideologue like Mao or Stalin, who ruled their empires for nearly 30 years. His famous statement that “it does not matter if the cat is black or white so long as it catches mice” made the Chinese reform work. The only problem with that quotation is that Deng said it at a Communist Youth League conference in 1962; therefore, it was not meant for a moment to be interpreted as the CPC’s attitude *after* 1989.^12^ Few storytellers care about such bibliographic detail, however.

Deng was behind some critical changes in the political system. Already in 1982, China imposed term limits on high-level political positions (including the president and the Party’s first secretary) and significant decentralization measures, which included more power for village and township governments and a substantial move towards some democracy at the local level (Oi 1999). The big test came in 1989. Deng had practically retired from day-to-day politics by then. His closest ally, Prime Minister Zhao Ziyang, was sympathetic to the student demonstrators and even tried to negotiate with them. The conservatives wanted to show force, but it was Deng who had to make the final choice. He opted for brutal force at Tiananmen Square. His “boss”, Zhao, lost, was sacked, and spent the rest of his life under house arrest. Deng did not stand behind him. Conservatives tried to use 1989’s political crisis to undo the reforms and return to the Maoist model. It was only after Deng’s *Southern Tour* in 1992 that the reform was back on track, and it was now officially equated with the transformation of China into a “socialist market economy” (Shi 1998, p. 6).

From Kornai, we knew that it was doubtful from the very beginning whether such a hybrid system could be sustainable without turning the political system from a communist dictatorship into some form of legal-rational authority (if we may use a Weberian term here). Our interpretation is that China for more than three decades after 1978 was cautiously building “capitalism from below”^13^—more and more capitalist and less and less just a hybrid political system. In 1983, the *rural system* of people’s communes was replaced by townships (Oi 1999). The reforms allowed certain *free market forces* to operate. The two measures together unleashed an economy based on rural family farming. As communist cadres and the urban population were increasingly becoming dissatisfied (Nee 1988), the reform was an attempt to pacify the local cadres by creating township and village enterprises (TVEs).^14^ The policy then gradually shifted—more forcefully after Deng’s Southern Tour—to cities and industry. Nevertheless, in the early stages of the reform, when the private sector was still discriminated against, private firms often were classified as collectives. That term remains in use today. The Statistical Office classifies firms as follows: state-owned, collective, cooperative, joint and limited liability share companies, private firms, funded by Hong Kong, Macao and Taiwan, foreign companies, and self-employed units. No wonder the statistical distinction between China’s private and public sectors is blurred (Kolodko 2018; Nuti 2019).

Similar to the agrarian reforms in Hungary from the mid-1960s onwards, agricultural prices were gradually deregulated. Production quotas were lowered and later eliminated. The result was a closing of the gap between urban and rural incomes. The big winners were the peasants. When China began reforms in 1978, it was still primarily a rural, agrarian country. To some extent, the nation’s extraordinary economic growth explosion can be attributed to its inexhaustible labor supply.^15^ Initially, the 1978 reforms may not have been much more than an attempt to moderate the Cultural Revolution’s extremes, and especially to solve the devastating but recurrent food shortages.^16^ That goal was reached by undertaking relatively minor adjustments, especially by shifting from agrarian communes to the household responsibility system.

Unlike many other countries (e.g., Russia or India), an ample “historical reservoir” of rural entrepreneurship is found in China. As Huang (2008, pp. 57–62) has pointed out, entrepreneurship in China had deep rural roots, not only in agriculture. Whyte (2009) also emphasizes that China had had centuries of extensive commercial development and intensive agriculture. Instant familiarity with markets exists among ordinary villagers (see also Rawski 2007, p. 103). Of course, one has to be careful in ascribing entrepreneurial success to such a historical reservoir, that is, to China’s cultural heritage. After all, since Max Weber, the received wisdom has been that Chinese culture in general, and Confucianism in particular, have been obstacles to modernization and entrepreneurship. But the rise of capitalism in East Asia and China’s economic growth during the past three decades does not necessarily exclude the Weberian argument. After all, Weber was interested in the origins of capitalism, and why capitalism emerged in the West and not in the Orient. The question to be posed now is this: can the elements of traditional culture be reassembled to fit the requirements of modernity (Peng 2005, p. 345)? The answer many scholars gave to that question was “yes” (Vogel 1991, pp. 92–101; Peng 2005; Whyte 2009).

The next stage was creating isolated *free-trade zones* attracting mainly small-scale Chinese capital from Hong Kong and Taiwan. After 1985, and especially after 1989, the reform began to shift from the countryside to the cities. The creation of private property rights in state-owned enterprise happened much later, but even when the enterprise sector was privatized to a large extent, the word “privatization” was never used in official documents. Instead, Chinese authors preferred to speak about *cross-border mergers and acquisitions,* a terribly misleading euphemism (Chen and Young 2010). In retrospect, it also is important to underline that the concept of private property was not incorporated into the Chinese constitution until 2004.

## The pendulum swings back

As we know from Freedom House data—and publications by Larry Diamond (2013, 2015, 2019) and others—the “third wave of democratization” (© Huntington) came to an end worldwide around 2005. Over the past 15 years, the democratization trend stopped and then reversed. The number of countries that became illiberal (“partially democratic”, “electoral authoritarian”, “non-electoral authoritarian”, or “unfree”) in Freedom House’s terminology has expanded. China, however, was capable of keeping her positive international image until very recently (although it was ranked as “unfree” by the Freedom House experts all along). Until 2020, the Chinese economy’s growth was stellar year after year. Chinese firms (though often state-owned enterprises) proved to be very successful on international markets, foreign direct investment (FDI) was pouring in and out of the economy on the order of hundreds of billion dollars annually, the names of Chinese billionaires filled the business columns of the world’s English-language newspapers, and so on.^17^ Those sources duly reported examples of China’s income inequalities, notorious corruption cases, deteriorating environmental conditions, and the like, but all such negative features fitted reasonably well into the general characterization of an illiberal, capitalist system (Zakaria 1997). On that basis, many Western commentators (e.g., Sachs 2005) anticipated that it was only a matter of time before China’s economic success would turn the country into a liberal democracy.

The fact that Chairman Mao’s gigantic poster has been displayed all along at Tiananmen Square and his photo is reproduced on the Renminbi banknotes of *all* denominations has not bothered foreign analysts. Those analysts also were inclined to neglect the unambiguous first articles of *The Constitution Law of People’s Republic of China* as well:

1. The People’s Republic of China is a socialist state under the people’s democratic dictatorship led by the working class and based on the alliance of workers and peasants.
2. The socialist system is the basic system of the People’s Republic of China. Sabotage of the socialist system by any organization or individual is prohibited.

Until President Xi Jinping elevated himself to the top of the political hierarchy, one could argue convincingly that despite the governing party’s official name (Communist Party of China, or CPC) and its insistence on standing for the cause of socialism (“The Party’s highest ideal and ultimate goal is the realization of communism”—as the CPC’s 2017 Constitution states in its very first paragraph), it was not the same party that existed at the time of Mao Zedong. Daniel A. Bell (2008) suggested half-jokingly that the CPC might be renamed as the Confucian Party of China, and he certainly had a point. The CPC’s historical trend moved away from emphasizing Marxism-Leninism-Maoism, especially class struggles. Moreover, Deng Xiaoping did place a great deal of weight on one of the central values of *Confucianism*, namely meritocracy; in the Hu-Wen era^18^ between 2003 and 2013, another Confucian idea, that of social harmony, was invoked rather often.^19^ For the distant observer, the CPC at the beginning of the 21st century would have more closely resembled the Kuomintang of 1950 than the CPC of 1978. In the academic world and the Western media, the same ideas were translated as *Chinese pragmatism* (Roland 2019).

For many years, János Kornai’s overall assessment was not very different from that of the Western academic mainstream. He admired many aspects of the reforms (e.g., sustained GDP growth, visibly fast technological modernization, the ending of the “one-child policy” in 2015,^20^ and state-supported massive enrollment of Chinese students at world-class Western universities). Like others, as a fact of reality, he also accepted the Communist Party’s monopoly (Farkas 2016, 2018). On one occasion, he used the term “quasi-communist party”, the members of which were allowed to participate in corrupt local businesses, including the so-called *princelings* (children of high-ranked cadres), who in that unique way have created their own stakes in the Chinese economy’s transformation.^21^ The predominant role of the CPC is *not* a very high price to pay to avoid a civil war, he asserted at that time. Furthermore, Kornai was pleased to acknowledge that, relative to Mao’s time, even the dictatorship’s brutality had softened considerably. In a 2014 paper published in Hungarian only, he still saw a “50–50” chance for “democratization of political institutions”, as opposed to the possibility of ruthless and naked repression. There, Kornai turned the customary argument in the opposite direction. The slowdown of Chinese GDP growth—which coincided with the aftermath of the 2008 international financial crisis—can lead to aggressive pro-growth government policies, to an increase in investment, and to cuts in wages and welfare spending. If that was the direction in which things would go, Kornai warned, tensions and protests could be met by more forceful retaliation, the country’s leaders might set out to incite nationalism or even try a foreign military adventure. But there was an equal amount of chance for a good outcome—continued democratization, welfare reforms, and the curtailment of corruption.

## Kornai’s U-turn

As discussed above, China was and frequently continues to be seen as a capitalist country that entered the market reform trajectory very early on. Whether that perception is correct depends on what we mean by reform and how we evaluate how far China has moved from the *classical system* of socialism (Kornai 1992).

To the surprise of many foreign observers, the consolidation of power in the hands of General Secretary and President XI Jinping (often described since 2013 as China’s “paramount leader” and “core leader” from 2016, and often called “Father Xi” in the party-controlled press) has led to a gradual rebuilding of a communist political system—not seen since the death of Mao in 1976. As of 2020, we observe more and more signs of Xi accumulating power in his own hands, of a developing personality cult and of the “selective criminalization” of his political opponents, clothed in the ideologically more acceptable garb of an anti-corruption campaign. In our interpretation, three things have been unfolding in parallel.

- *Firstly*, Xi consciously and determinedly has been pushing the country towards a one-man dictatorship justified by the ideological legacies of Mao and Deng.^22^ In October 2017, the party’s National Congress called for a constitutional amendment to repeal term limits and other important safeguards adopted in 1982. Unsurprisingly, all of the proposed changes were built into the Constitution in March 2018. By repealing the presidential term limit, that constitutional amendment has made it possible for Xi Jinping to remain China’s supreme leader as long as he so desires.
- *Secondly*, given the importance of China’s emergence as a global superpower, the outside world gets more and more detailed information about controversial issues that were already known, but few outsiders care about their details and implications.^23^ The *hukou* system is a good example. People who were born in the countryside and who now live in Beijing and other major cities are treated as second-class citizens. Several hundred million rural Chinese who are urban guest workers do not even get basic government provisions for their livelihoods because of how the *hukou* system ties them to their places of permanent residence, although in sectors and locations where labor shortages are endemic, the implementation of *hukou* rules became less severe.^24^
- *Thirdly*, new facts emerge that were almost entirely hidden behind the veil of secrecy. Since 2017, for example, numerous reports have appeared about ethnic Muslim members of the Uyghur and Kazakh communities in the eastern part of the country, who were detained in extrajudicial “re-education camps”. Estimates from 2018 placed the number of detainees in the hundreds of thousands. China also continues to follow very oppressive policies against Tibet. In other words, Xi tries to transform a multi-ethnic, multilingual China into an ethnically, linguistically homogeneous nation state.^25^ Another frightening new development is the so-called Social Credit System, a de facto blacklist. By the end of 2020, it is intended to standardize and centralize administrative assessments of citizens’ and businesses’ economic and social reputations. The social credit initiative calls for the establishment of a unified record system for individuals, businesses, and the public sector, to be tracked and evaluated for political trustworthiness.

A few weeks *before* the above-mentioned constitutional reform, Kornai (2018) gave a speech devoted partly to China at a two-day Budapest conference organized to celebrate his 90th birthday in February 2018. China is not going in the right direction, the dictatorial features of the system become stronger and stronger, he stated. Speaking about the risks emerging from the country’s lasting dynamic growth, the sheer size of the Chinese economy and the erosion of multilateral cooperation, he said the following.

The official Chinese ideology is very much influenced by nationalistic ideas. Now, if China were the only nationalist power in the world, then one can think about isolation. But that is not the case. There are other giants, which are also nationalists. There is the USA, where the President is announcing ‘America first’. Not the globe first, not the international community, not the future of the international community first, but America first. And then we have Russia, where the leadership again is explicitly and emphatically nationalistic. (Kornai 2018, p. 62)

After alarming his Hungarian audience, Kornai (2019a) shocked the international community 14 months later in the form of a *Financial Times* (FT) op-ed on 11 July 2019.^26^ The two main messages of that short article were built into its title (“Economists share blame for China’s ‘monstrous’ turn”) and its subtitle (“Western intellectuals must now seek to contain Beijing”). Kornai’s words were unusually harsh, self-critical and normative. He recalled his own personal direct contribution to Chinese economic reforms and the fact that he (and others, of course) helped the Chinese communist leadership to gain worldwide sympathy among leading politicians and intellectuals for the economic successes, while they all closed their eyes to China’s serious curtailment of human rights. Kornai wrote, “We, Western intellectuals dealing with China, are—perhaps with a few exceptions—the Frankensteins of our time. Many of us already bear moral responsibility for not protesting against the resurrection of the monster. And there are those whose responsibility goes much further, because they have taken on an active role as advisors. I include myself here.” In an unusual gesture, Kornai directed advice to policymakers and public intellectuals worldwide and made historical reference to the beginning of the Cold War: “Decades ago, in the context of the threatening US-Soviet confrontations, a high-ranking American diplomat, George Kennan (1947) summarized what should be done with the expression ‘containment’. Thus far and no further! Or more precisely: no further in this direction! What has happened already cannot be undone. But here we must stop, and we must take far more care to avoid carrying on in the role of Frankenstein.”

This short piece in *The Financial Times* (and its Hungarian translation) generated echoes both in Hungary and abroad that, in turn, motivated Kornai to write a longer and more detailed exposition of his views.^27^

Since the publication of the FT article, the situation has changed profoundly. In the September 2019 issue of *Foreign Affairs*, the leading US magazine for analysis of and debate about foreign policy, five lengthy papers were devoted to the assessment of President Xi’s policies and the risks emanating from them. One of those papers (Westad 2019) was built entirely on the relevance of Kennan’s (1947) paper, exactly aligning with Kornai’s arguments summarized above. That alignment was, of course, partly, but not entirely, coincidental. It seems that foreign policy analysts grasped the significance of the recent shifts in China more quickly than mainstream economists did. Seven months later, George Soros (2020) alarmed the European Union. He published an op-ed (under the telling title “Europe Must Recognize China for What It Is”) containing words very similar to those written by Kornai: “Neither the European public nor European political and business leaders fully understand the threat presented by Xi Jinping’s China.”

Let us summarize our analysis thus far. Ever since General Secretary of the CPC, Xi Jinping, became president, China had been turning more and more towards authoritarianism. Since 2013, Xi has been ruling like a dictator, and one even could begin to wonder if it really is a capitalist economy, or if it rather is what the CPC’s constitution claims the country to be: a “socialist market economy” with Chinese characteristics. Moreover, it is noteworthy that the official Chinese document released after Xi was elected to the presidency by the 13th National People’s Congress did not mention the word “market” at all (Xinhua 2018). When a report published by the state-owned news agency summarized the essence of “Xi Jinping[’s] Thought on Socialism with Chinese Characteristics for a New Era”, a long phrase that had been added to the country’s amended constitution, eight fundamental issues and 14 fundamental principles were listed. In the two-page-long text, the word “socialism” appears seven times, but the words “market”, “ownership” or “property” were not even mentioned on a single occasion.^28^

## How to delineate capitalist and socialist systems?

One of the authors of the present paper, Ivan Szelenyi (2010) reviewed the literature originating from China and claiming that China is “market socialism”. While like Kornai he also perceived China to be on its way from communism to capitalism, he drew attention to the literature on the “market socialism” thesis and advised Western scholars to be attentive to the socialist features of post-reform China, proposing that China—straddling communism and capitalism—could be seen as a hybrid economy. However, in a more recent contribution (Szelenyi and Mihályi 2020a, b), we changed our minds because we saw China reverting increasingly to *communist dictatorial* policies.^29^

Kornai, in one of his recent, extensive writings, made an instructive distinction between the *primary and secondary characteristics of economic systems.* We adopt those two categories without modification, and then supplement them with a third list of additional characteristics.

So, according to Kornai (2016), the three primary characteristics of socialist systems are the:

1. political monopoly of a one-party state that legitimates itself with the ideology of Marxism-Leninism;
2. means of production controlled exclusively by public ownership;
3. dominant form of coordination that is bureaucratic rather than market-based.

Until 1989, that three-dimensional definition was sufficient. But the world is messy and keeps on changing. No one can say, in 2020, that China meets *all* three criteria fully (see Block A in Table 1), or stating the opposite, that *none* of the three criteria has any relevance, because China switched to the capitalist model. No doubt exists, however, that China does have widespread private ownership and several building blocks of a market economy. Nevertheless, China and her Asian satellite countries are functioning under the control of one-party systems, the ruling parties legitimate themselves in Marxist-Leninist terms, the scope of private property is limited in many ways, and the statist sector of the economy—especially in finance—still plays critical roles.

**Table 1:** The main characteristics of the Chinese society and economy, as of 2020

	Similar to capitalist systems		Similar to socialist systems	
	Block A: Primary characteristics^a^			
1	The ruling political group ensures the dominance of private property and market coordination	No	Yes	The ruling political group (the Communist Party) enforces the dominance of public ownership and bureaucratic coordination
2	Dominant form of property: private ownership	Partly	Partly	Dominant form of property: state ownership
3	Dominant form of coordination mechanism: market coordination	Partly	Partly	Dominant form of coordination mechanism: bureaucratic coordination
	Block B: Secondary characteristics^a^			
4	Surplus economy. The buyers’ market is the dominant state of the market for goods and services	Yes	No	Shortage economy. The sellers’ market is the dominant state of the market for goods and services
5	Labor surplus is the dominant state of the labor market	Yes	No	Labor shortage is the dominant state of the labor market
6	Fast technical progress; the system often generates revolutionary innovations	Partly	Partly	Slow technical progress, the system rarely generates revolutionary innovation
7	High income inequality	Yes	No	Low income inequality
8	Hard budget constraint for organizations in a quite broad sphere	Yes	Yes	Soft budget constraint for organizations in a quite broad sphere
9	Direction of corruption: it is mostly the seller who bribes the buyer	Yes	No	Direction of corruption: it is mostly the buyer who bribes the seller
	Block C: Further characteristics^b^			
10	Liberal state. Multi-party elections matter, checks-and-balances and free media	No	Yes	Homogeneous party-state structure, no free and fair multi-party elections. Media censorship
11	The power of prime ministers and presidents is limited by rules and competitions	No	Yes	One-man rule. Cult of personality
12	Secure property rights	No	Yes	Patrimonial property rights, selective criminalization of oligarchs
13	Political parties are not allowed at workplaces	No	Yes	The communist party is present in all workplaces (including the private sector)
14	Individualist culture	No	Yes	Collectivist culture
15	Fluctuations of the investment rate (business cycle)	No	Yes	The share of investments is very high in GDP
16	Free movement of labor (including migration)	No	Yes	Limited freedom of migration (even within the country)
17	Full convertibility of the currency	No	Yes	Limited convertibility
18	Liberalized foreign trade, strong export orientation	Yes	No	Closed economy, autarchy
19	Predominantly liberalized land market	No	Yes	All forms of land are in public ownership
20	Foreign policy may or may not be a priority	No	Yes	Ideology-based foreign policy is a top priority

^a^Based on Kornai (1992, 2016)

^b^Authors’ own compilation

Our opinion is based partly on Chinese sources. While China specialists—like Philip Huang (2012) on the far left, Yasheng Huang (2008) on the classical liberal side, and Fan et al. (2019) more recently—see China as an integrated market, they all doubt that private property became dominant; hence, for them, the economy is *not* capitalist. Huang estimates that by the end of the first decade of the 21st century, 70% of non-agrarian products were still being produced by the state sector, while the IMF put the same figure at just 30%. Nevertheless, both Philip Huang and Yasheng Huang agree that the interactions between publicly owned firms are market regulated. Yasheng Huang claims that enterprise privatizations mainly took the form of various state-owned banks and corporations buying up the shares of publicly owned firms that had been put on the stock exchange. In the enterprise sector, individual private ownership exists, but it remains of secondary importance. Philip Huang (2012) also claims that the overwhelming majority of the 50 or so Chinese multinational corporations listed in the *Forbes 500* were, in fact, state-owned. If that number is correct, then China created something like what a Hungarian reform-economist, Márton Tardos (1975), called a “network of holding companies” nearly a half-century ago.

We believe that at least three reasons can be found to explain why arguments that China is socialist should be seriously considered.

a China has been slow to recognize private property rights and it often is doubtful whether what is being called private property actually is private.
b The Chinese state is involved so intimately in economic processes that it arguably is beyond the roles usually assigned to a “developmental state”, even in its East Asian variant.
c The most obvious reason: China is a one-party state wherein communist ideology (even if the emphasis increasingly is on nationalism) legitimates the party.

Let us consider the three arguments in the framework of Table 1’s 20 strong assertions. As Martin Whyte (2009) and Huang (2008, p. 31) pointed out more than 10 years ago, secure *property rights* usually are regarded as a major precondition for capitalism and dynamic economic development, yet China has proven to be an outlier in that respect.^30^ The national Property Law was passed only in 2007 (before 2007, various limits were imposed on the number of employees domestic firms could hire), and even that law was rather restricted in scope. As such, China diverged radically from Europe’s post-communist countries, which, as far as property rights were concerned, followed the Washington consensus cookbook rather closely. Most of those countries privatized early and fast; priority was assigned to creating identifiable owners even if that meant transferring state property into the hands of former communist *nomenklatura*^31^ (which happened especially in Russia) or to foreign multinationals (which happened especially in Eastern and Central Europe). China followed a dual-track approach: until the late 1990s, it rejected the privatization of large state-owned enterprises (SOEs), while allowing foreign capital into the country in the form of “greenfield” investments and permitting the launching of *de nouveaux* domestic private firms, albeit with some limits. It is very difficult to measure personal wealth accurately in China. Some billionaires may hide it. Others, like princelings (especially the family of President Xi), may oversee large companies that formally are SOEs but could be de facto private businesses owned by their princeling-managers.

The private ownership of agricultural land remains restricted or, to be more precise, banned constitutionally. Even though family farming was restored during the early 1980s, peasants did not receive titles to the lands they till. They lease land from the villages; the terms of the leases were gradually extended up to 30 years in 1984. Although leases had practically become indefinite by October 2008, still no titles were granted to private individuals. As Nuti (2019) noted fittingly, the system works like the *arenda* system that spread during the USSR’s New Economic Policy (NEP) of 1921–1926. Some commentators regard the October 2008 resolution of the party’s Central Committee as a landmark event (Li 2009a) since it gave full rights to farmers to subcontract, lease, exchange, or swap their land-use rights.^32^ Optimists expected that the resolution would enable retiring farmers to purchase homes in urban areas, that it would improve capital flows into rural areas, and—especially during the 2008 global financial crisis when China’s exports dropped sharply—that it would increase domestic production and help China to change course from export-led industrialization to an economic growth path driven by domestic consumption. Li Cheng saw the new land reform as a step towards eliminating the *hukou* system, which had turned those who held rural *hukou* (especially migrant workers in cities with rural registration) into second- or third-class citizens. Land reform, it was hoped, might boost the income and consumption of former rural *hukou* registrants.

Let us now refer to Block B in Table 1, the six *secondary* characteristics of idealized economic systems—i.e., capitalism versus socialism. As we have noted already above, the points included there are taken directly from Kornai (2016). Today, China certainly is not a shortage economy, and direction of corruption is similar to that of market economies: it is mostly the seller who bribes the buyer (e.g., in the course of public procurement tenders). Surplus dominates the labor market and income inequality is high. Thus, in those four key aspects, China displays market economy features. The question of hard or soft budget constraints (SBCs) is difficult: many examples of both can be found. SOEs and state-run banks enjoy the benefits of SBCs, and even genuine private companies are bailed out when subvention seems essential for one political reason or another (Nuti 2019). It is not easy to assess the pace of technological change, either. In many ways, Chinese firms are extremely efficient, but at the same time, it remains true that revolutionary innovations are few and far between. Nonetheless, at least one Chinese firm (Huawei) ranks at the top of the international innovation curve, and the present COVID-19 crisis provides a chance for Beijing to come out first with an effective vaccine. The jury is out.

In Block C, we list 11 further characteristics. Here we touch on certain spheres that were never close to Kornai’s research agenda (e.g., foreign trade, monetary policy, and agriculture). We conclude that on 10 dimensions of the 11 dimensions listed, present-day China seems to closely resemble the well-known, classical socialist model. Looking at the dimensions of the analysis one by one, our judgments are not fully justified, but we believe that the overarching conclusion of Block C is convincing: China has kept many of the features of its pre-1989 version of communism.

Since 1989, Kornai has never changed his position on the political evaluation of the Chinese system. While he noted the gradual, but significant, softening of the CPC’s power monopoly, he never questioned that China is a dictatorship. Yes, it was, but it was shifting for a while towards a “non-electoral autocracy”, with term limits imposed on presidents, with significant powers allocated to the Politburo’s rather diverse executives, and some liberal legislation at least for foreign investors (and some reasonably competitive elections at the local level). We also refer to a vague, nonetheless very forceful definition Mao Zedong gave to characterize his system: “politics in command”.^33^ Obviously, for the Chinese leader back then, the slogan meant the Marxist-Leninist interpretation of politics. Not all dictatorships are communist, but Mao’s “politics in command” likewise is a good self-characterization of all other types of despotism. For example, in Russia, Iran, or the Gulf monarchies, different ideologies (ethno-nationalism, tribalism, Christian traditions, Islam, or some combination of them) are equally suitable for legitimizing dictatorial regimes (Table 2).

**Table 2:** Varieties of capitalism in comparison with the socialist systems in the 21st century. *Source*: Authors’ compilation

	Capitalist systems	Socialist systems
1	Liberal states: Elections matter	x
	(a) Liberal market economies (as little state intervention as possible) E.g., Australia, Czech Republic, Hungary (until 2010), UK, and USA	
	(b) Coordinated market economies, conservative version (with targeted social policy interventions) E.g., Post-WWII Germany	
	(c) Coordinated market economy (Social-democratic version) E.g. Scandinavian countries	
2	Illiberal democracies: Ethno-nationalist policies, but elections matter E.g., Hungary (since 2010), India (under Modi), Japan, Poland (since 2015), Russia (under Yeltsin), Singapore, Taiwan, and Turkey	x
3	Dictatorship or non-electoral autocracies: Politics and ideology trump economic rationale + there are no elections, or they do not matter E.g., China (1978-2012), Gulf monarchies, Iran, Russia (under Putin), several post-Soviet republics	Dictatorship: Politics is in command + Elections do not matter E.g., China (since 2012), Cuba, Laos, North Korea, and Vietnam

## How to delineate illiberalism from dictatorship?

In our vocabulary, “illiberalism” and “dictatorships” have different meanings. Illiberalism is a project to create an authoritarian regime, with few limits on the executive branch, but in illiberal regimes elections still take place, the judiciary still has some autonomy, and the media is somewhat free. None of those meanings really hold for China today. Questioning the logic of the Varieties of Capitalism (VoC) paradigm, as it was originally formulated by Hall and Soskice (2001), we see three different versions of capitalism: liberal states, illiberal states, and capitalist dictatorships. As we have tried to argue throughout this paper, socialist systems currently take only one form: dictatorship And that is the box into which—despite their different self-categorizations—the Kingdom of Cambodia, the Republic of Cuba, the Lao People’s Democratic Republic, the Democratic People’s Republic of Korea, and the Socialist Republic of Vietnam fit. The Peoples’ Republic of China (PRC) increasingly fits, too, although between 1978 and 2013, the regime in Beijing drifted slightly towards a non-electoral autocracy. That drift was altered substantially when President Xi took power. It may sound strange and controversial, but all in all, as of today, we must agree with the ideological self-labelling of the CPC: *China is socialism with Chinese characteristics*.

The classification of China is enormously important. Socialist countries are all dictatorships driven by Marxist-Leninist, one-party states and communist ideologies. If China is considered to be a capitalist dictatorship, then only a few unimportant countries like Cuba and North Korea would remain in the [what?] box. In other words, if our analysis is valid, then Fukuyama (1989) was entirely right: history has ended. On the other hand, if we accept that China (and its four Asian satellite partners + Cuba) are communist systems, then history has not ended in the way Fukuyama interpreted it.

Let us close our paper with a direct quote from Kornai’s seminal writing, when he first coined the supermarket metaphor, with which we started our present paper.

History does not provide such supermarkets in which we can make our choice as we like. Every real economic system constitutes an organic whole. They may contain good and bad features, and more or less in fixed proportions. The choice of system lies only among various ‘package deals’. It is not possible to pick out from the different ‘packages’ the components we like and to exclude what we dislike. It seems to me that it is impossible to create a closed and consistent socioeconomic normative theory which would assert, without contradiction, a *politico*-*ethical value system* and would at the same time provide for the *efficiency* of the economy. (Kornai 1980, pp. 156–157; our emphasis)

We agree with Kornai, with one qualification. China was on its way to market capitalism with the possibility of turning eventually into a liberal democracy. The road was rocky, with many back-and-forths. But the shift to liberal democracy did not happen. The 1989 massacre at Tiananmen Square, approved by Deng, was a more alarming setback than contemporary Western observers realized. Today, we are much like Kornai, increasingly skeptical about what has been happening in China for the past eight years. Kornai did a U-turn in his theorizing about China, but what is more important, China seems to be taking a U-turn back to the pre-1978 epoch.

## Footnotes

1 Taking our lead from Kornai (1992, 2016), we use socialism and communism as synonyms throughout this paper.

2 The term comes from Max Weber’s “elective affinity” (*Wahlverwandtschaften*; think about the plural in the German expression!), but for Weber that term was not supposed to be a causal relationship. It does not imply that no choices are available; it means only the likelihood that one system characteristic will match another. In the early formulation of Kornai, “affinity” read like an inevitable, causal relation. To put it another way, the question is whether we have a set *prix fixe* menu or *á la carte*. Well, some restaurants offer fixed menus with multiple choices of main dishes. China is an excellent example of a set menu with multiple main dish options. Kornai—as a good social scientist and faced with the realities of the Chinese economy and society—adjusted his theoretical frame, as we will show in this paper.

3 In his memoir, Kornai (2006, p. 282.) recalled that he had used the supermarket metaphor for the first time in the Q&A part of his 1979 Geary Lecture in Dublin. In the edited version of the lecture, that point was already elaborated in more detail at the essay’s end (Kornai 1992).

4 For a similar position see Bell (2008).

5 Before the U-turn, Nee and Opper (2007) called China “politicized capitalism”. Huang (2008) wrote about “capitalism with Chinese characteristics”.

6 A direct quote from the communique of the Chinese foreign ministry on the content of President Xi’s phone conversation with US President Donald Trump on 7 February 2020.

7 Fukuyama was extrapolating the analysis of his mentor at Harvard, Samuel P. Huntington, who wrote an agenda-setting work on what he called “the third wave of democratization”. Huntington’s point of departure was the 1974 anti-fascist revolution in Portugal and the death of Spain’s General Franco in 1975. See Huntington (1991), although by 1996 Huntington’s optimism was fading and he began to see a new world disorder, based not on competition between capitalism and socialism, but between Western liberal democracy and other illiberal systems not necessarily conducive to capitalist development.

8 By 2020, only North Korea and to some extent Cuba are communist in the classical sense. If China before Xi was authoritarian capitalism, that term also fits well in the cases of Cambodia, Laos, and even more so Vietnam.

9 In an interview with the editor of *Encyclopedia Britannica*; see Yu (2005).

10 Gao (2008) claims that Deng was never a Marxist, but rather a pragmatic Chinese patriot.

11 Although he kept the title of “deputy”, Deng was never prime minister, president, or first secretary of the Party.

12 See Chambers Dictionary of Quotations (1993 p. 315), referenced in https://en.wikiquote.org/wiki/Deng_Xiaoping, last visited 7 February 2020.

13 For two slightly different interpretations of the term, see Eyal et al. (1998) and Nee and Opper (2012).

14 Mostly a planned transitory process aimed towards market-conforming, conventional firms. Since the mid-2000s, the Chinese government no longer provides statistical data for TVEs as a separate independent sector.

15 Sachs (2005, p. 158). Labor reserves remain abundant. According to the World Bank, 18% of China’s 2017 working population remained employed in agriculture.

16 In the 20th century alone, China has suffered six major famines, resulting in the loss of millions of lives.

17 The latest *Forbes* list of billionaires contains five Chinese entrepreneurs among the globe’s top 40, the richest being 48-year-old media magnate Ma Huateng, with USD 39 billion in assets ranking in 20th place. See https://www.forbes.com/billionaires/list/;#version:static; last visited 28 January 2020.

18 The phrase is named after the Party’s General Secretary and President Hu Jintao and Government Premier Wen Jiabao, who were considered to represent the fourth generation of Chinese leaders. They were viewed, at least ostensibly, as more reform-oriented and more open-minded than the leaders of the third generation. In Chinese ideological publications, the dominant political slogan of the two was called the Scientific Development Concept.

19 Along the same lines, Kolodko (2018) characterized China as a “hybrid system in the form of socialist capitalism or … capitalist socialism …, a sort of *Chinism*”.

20 Actually, the “one child policy” was introduced in 1979 at the early stages of the Deng reforms.

21 At one time, 28% of the members of the Politburo were princelings (Li 2009b).

22 Deng’s name is mentioned explicitly in the Preamble of the Constitution together with Marxism-Leninism, Mao, and Xi. That mention is intriguing, since Deng was not a Maoist. But Xi must pay respect to Deng in the same way he had to acknowledge Mao’s historical importance. In the Confucian Chinese culture, you *must* show respect to your predecessors irrespective of your personal opinion or the facts.

23 *The Economist*, for instance, has extended its coverage of China significantly. In every issue, readers find a China section containing two or three well-researched articles. The same holds true for daily issues of *The New York Times*.

24 Although the origins of China’s *hukou* system date back to ancient times, the system in its current form came into being in 1958. A similar registration system was known in the USSR from 1932 until the fall of communism. In present times, an analogous household registration system exists within the public administration structures of Japan (*koseki*), Vietnam (*hộ khẩu*), and North Korea (*hoju*). South Korea’s *hoju* system was abolished only in 2008.

25 The relationship between communism and nationalism is very complicated. In the 19th century, communism was as internationalist ideology. In 1924, Stalin declared the principle of “socialism in one country” and his system took a nationalist turn, but it still retained an internationalist mission. Soviet involvement in Africa or Latin America was, in part, a Russian imperial project, but it also was driven in part by the dream of turning the world communist. Ethno-nationalistic movements in countries like Yugoslavia or Czechoslovakia were kept under strict control. A sharp difference exists between Stalin and Putin in this respect. Putin follows the Russian imperial project. While Mao also kept the “empire together”, Xi has worldwide imperialist ambitions. Donald Trump’s aim is to “make America great again”. Putin and Xi likewise want to make Russia and China “great again”.

26 Kornai’s piece was translated into Chinese in July by a Hong Kong newspaper and into Vietnamese. An influential multilingual Chinese newspaper gave an extensive summary of the FT essay (Street 2019). The latter one, to the best of our knowledge, was disseminated only on the Internet.

27 The two opinion pieces were re-published in an integrated form in the English-language academic quarterly *Acta Oeconomica*, published under the editorial control of Kornai (2019b).

28 As long as the official Chinese documents do not embrace the term “market economy” without reservation, Beijing cannot convincingly protest the legal treatment of the country by the World Trade Organization (WTO), in which China has been a member since 2001, but not recognized as a “market economy”. Without a WTO ruling in Beijing’s favour, the EU and the United States can keep imposing duties on cheap imports from China while disregarding its claim that they are priced correctly.

29 Throughout the present section—and in Table 1 in particular—we make a clear distinction between illiberal systems and dictatorships on the basis of social and economic considerations, but we are aware that Freedom House and many political scientists (e.g., Bieber et al. 2018) define illiberalism as a hybrid of democracy and dictatorship.

30 See also Oi (1999) on property rights issues.

31 The term *nomenklatura* (originally a value-free Russian word, borrowed from Latin, meaning a list of names) was used widely in the Soviet Union. In all former socialist countries (including China), it meant a category of people who held various key administrative positions in the bureaucracy, running all spheres of society: government, industry, agriculture, academia, and so on, whose positions were granted only with the approval of the country’s Communist Party. Virtually all members of the nomenklatura belonged to the Communist Party. Nomenklatura is a concept similar to the western “Establishment”, holding or controlling both private and public powers (e.g., media, finance, trade, industry, state, and other institutions). In other words, nomenklatura is a broader concept than “elites”. For the Chinese context, see Xu (2019).

32 A recent empirical study, however, reports that rural land rights confirmation had significant and positive effects on the likelihood and amount of transfer-out land at the 5% significance level, but the effect on transfer-in farmland was insignificant. See Xu et al. (2017).

33 In a widely publicized talk during the Cultural Revolution, when Mao and Lin Piao “reviewed 600,000 revolutionary students and teachers and Red Guards” marching at Tiananmen Square, the former gave the following instruction to his closest followers: “You should put politics in command, go to the masses and be one with them and carry on the great proletarian revolution even better.…” See the *Peking Review*, Vol. 9. #47, Nov. 18, 1966. https://www.marxists.org/subject/china/peking-review/1966/PR1966-47b.htm, Last visited: 29 January 2020.
